# Reoperations after fusion surgeries for degenerative spinal diseases depending on cervical and lumbar regions: a national database study

**DOI:** 10.1186/s12891-021-04491-3

**Published:** 2021-07-10

**Authors:** Moon Soo Park, Young-Su Ju, Seong-Hwan Moon, Young-Woo Kim, Jong Ho Jung, Jung Hyun Oh, Chi Heon Kim, Chun Kee Chung

**Affiliations:** 1 Department of Orthopaedic Surgery, Hallym University Dongtan Sacred Heart Hospital, Medical College of Hallym University, 7, Keunjaebong-gil, Hwaseong-si, Gyeonggi-do 18450 Republic of Korea; 2 Department of Occupational and Environmental Medicine, National Medical Center, 245, Eulji-ro, Jung-gu, Seoul, 04564 Republic of Korea; 3 Department of Orthopaedic Surgery, Yonsei University College of Medicine, 50-1 Yonsei-ro, Seodaemun-gu, Seoul, 03722 Republic of Korea; 4 Department of Orthopaedic Surgery, Hallym University Sacred Heart Hospital, Medical College of Hallym University, 22 Gwanpyeong-ro 170 beon-gil, Dongan-gu, Anyang-si, Gyeonggi-do 14068 Republic of Korea; 5 Department of Neurosurgery, Seoul National University Hospital, 101, Daehak-ro, Jongno-gu, Seoul, 03080 Republic of Korea; 6 Department of Neurosurgery, Seoul National University College of Medicine, 103 Daehak-ro, Jongno-gu, Seoul, 03080 Republic of Korea; 7 Neuroscience Research Institute, Seoul National University Medical Research Center, 103 Daehak-ro, Jongno-gu, Seoul, 03080 Republic of Korea; 8 Clinical Research Institute, Seoul National University Hospital, 101, Daehak-ro, Jongno-gu, Seoul, 03080 Republic of Korea; 9 Department of Brain and Cognitive Sciences, Seoul National University, 1 Gwanak-ro, Gwanak-gu, Seoul, 08826 Republic of Korea

**Keywords:** Spondylosis, Fusion surgery, Reoperation, Nationwide database

## Abstract

**Background:**

Reoperation is one of the key factors affecting postoperative clinical outcomes. The reoperation rates of cervical surgeries might be different from those of lumbar surgeries due to the anatomical and biomechanical differences. However, there has been no study to compare the reoperation rate between them. The purpose is to compare reoperation rates after fusion surgeries for degenerative spinal diseases depending on the anatomic region of cervical and lumbar spines.

**Method:**

We used the Korean Health Insurance Review & Assessment Service national database. Subjects were included if they had any of the primary procedures of fusion combined with the procedure of decompression procedures under the diagnosis of degenerative diseases (*n* = 42,060). We assigned the patients into two groups based on anatomical regions: cervical and lumbar fusion group (*n* = 11,784 vs 30,276). The primary endpoint of reoperation was the repeat of any aforementioned fusion procedures. Age, gender, presence of diabetes, associated comorbidities, and hospital types were considered potential confounding factors.

**Results:**

The reoperation rate was higher in the patients who underwent lumbar fusion surgery than in the patients who underwent cervical fusion surgery during the entire follow up period (*p* = 0.0275). A similar pattern was found during the late period (*p* = 0.0468). However, in the early period, there was no difference in reoperation rates between the two groups. Associated comorbidities and hospital type were noted to be risk factors for reoperation.

**Conclusions:**

The incidence of reoperation was higher in the patients who underwent lumbar fusion surgery than those who underwent cervical fusion surgery for degenerative spinal diseases.

Reoperation is one of the key factors affecting postoperative clinical outcomes.

The reoperation rates of cervical surgeries might be different from those of lumbar surgeries due to the anatomical differences.

However, there has been no study to compare the reoperation rate between them.

In a national population-based cohort study, the incidence of reoperation was higher in the patients that underwent lumbar fusion surgery than those which underwent cervical fusion surgery for degenerative spinal diseases.

To the best of our knowledge, this study represents the first population-based analysis of the reoperation rates after fusion surgeries according to cervical and lumbar regions.

## Background

Concomitant cervical and lumbar surgeries are not uncommon [1–3]. Jacob et al. have found that in a study population of 200 patients who underwent cervical spine surgery, thirty-one percent required additional surgeries in the lumbar spine [1]. The patients who underwent concomitant cervical and lumbar surgeries had satisfactory clinical results after the operations [2, 3]. The patients are likely to have concomitant cervical and lumbar surgeries due to the advance of diagnostic modalities and the aging society.

Reoperation is one of the key parameters showing postoperative clinical outcomes. The reoperation rate after fusion surgeries for lumbar degenerative diseases varied from 10.3 to 19.3% depending on the definition of reoperation, the follow-up period, or surgical procedures [4–6]. In the case of cervical degenerative diseases, reoperation rates after fusion surgeries were found from 4.8 to 15% [7, 8].

The degeneration of intervertebral disc is the main pathogenesis of spinal diseases and the disc degeneration in the cervical spine is correlated with that in the lumbar spine [9–11]. The degeneration requires surgical procedures not only for cervical spine but also for lumbar spine [12]. In contrast, the reoperation rates of lumbar fusion surgeries might be different from those of cervical fusion surgeries due to the anatomical and biomechanical differences. However, to the best of our knowledge, no study has evaluated the difference in reoperation rates between the two groups. It might be due to the difficulty to compare the reoperation rates because of a relatively low incidence of reoperation after fusion surgeries. National population-based databases provide a large cohort that may help overcome this challenge and a complete follow-up of reoperations without the follow-up loss, even after the patients were discharged from the hospital.

The purpose is to compare reoperation rates after fusion surgeries for degenerative spinal diseases according to cervical and lumbar regions with national population-based databases. The hypothesis is that the reoperation rates may be different between the two regions due to the different anatomical and biomechanical features.

## Material and methods

Since previous studies are designed with the most effective study design for the elucidation of the reoperation rate after surgeries [13, 14], we have applied the designs, data source, and surgical indications of the previous studies to the current one.

### Data source

The Korean Health Insurance Review & Assessment Service (HIRA) is a national database, which has a prospectively collected set of data. The data have information of roughly 51 million patients in the Republic of Korea and contain all inpatient and outpatient data reported according to diagnosis and procedure codes. The diagnosis codes are standardized according to the Korean Classification of Disease, 6th version, which follows the International Classification of Disease, 10th edition (ICD-10).

### Study population selection and design

We searched the HIRA national database to identify patients with a primary diagnosis of cervical spondylosis including cervical radiculopathy, myelopathy, and so on (diagnosis codes: M471, M472, M500, M501, M502, M503, M508, M510, M519, M541, M542, G951, G952, G958, G959. G992) or lumbar spondylosis including lumbar radiculopathy, spinal stenosis, spondylolisthesis and so on (diagnosis codes: M253, M431, M478, M479, M480, M511, M532, M545, M546, M548, M549, M992, M995).

The subjects were included if they had any of the following procedures of anterior or posterior fusion with the following procedure of decompression between January 1, 2012, and June 30, 2017: cervical anterior fusion (procedure code: N2463), cervical posterior fusion (procedure code: N2469), and lumbar anterior fusion (procedure code: N0466, N1466), lumbar posterior fusion (procedure code: N0469, N1460, N1469, N2470). The decompression procedure includes cervical discectomy (procedure code: N1491), cervical laminectomy (procedure code: N1497, N2497), cervical corpectomy (procedure code: N0451), lumbar discectomy (procedure code: N1493), and lumbar laminectomy (procedure code: N1499, N2499). The patients’ resident identification numbers were encrypted for privacy.

**Fig. 1:**
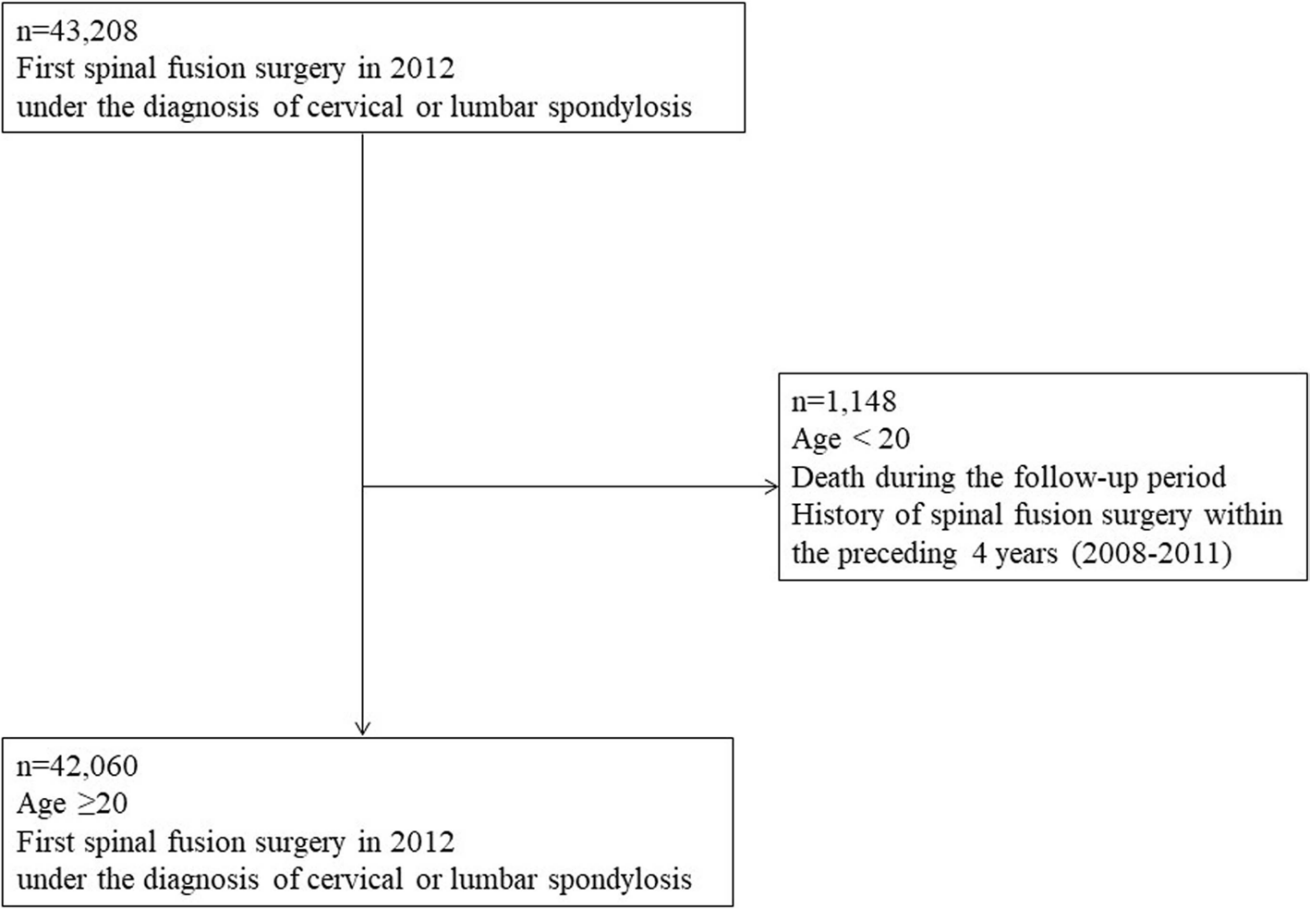
Cohort definition

A total of 43,208 patients, that under the diagnosis of spondylosis and underwent cervical or lumbar fusion surgeries in 2012, were selected from the cohort of patients (Fig. 1). Patients younger than 20 years old were excluded because we had intended to elucidate the reoperation rates after fusion surgeries for degenerative spinal diseases. Those who died during the follow-up period (causes of death were not recorded) were excluded. Patients were also excluded if they had a history of spinal surgery within the preceding 4 years (2008–2011) because the previous study to evaluate reoperation rates of cervical spine surgeries had revealed that the reoperation rates according to the surgical types were different until the follow-up of 4 years [13] and we had intended to minimize the effect of spinal surgeries on reoperation rates. The final study population of the patients who underwent fusion surgeries in 2012 was 42,060. All patients included in the study cohort were evaluated during the follow-up period of four and a half years between January 1, 2013, and June 30, 2017.

The subjects were divided into two groups based on the anatomical region where they had the index procedure: cervical or lumbar fusion group. Our goal was to determine changes in the reoperation rates over time and to compare the reoperation rates between the above-mentioned anatomical regions while confounding variables were adjusted.

### Surgical indications

In Korea, nearly all hospital follows the requirements of surgical treatments of the Korean National Health Insurance Corporation for reimbursement. In the case of anterior cervical decompression combined with fusion for cervical radiculopathy, these regulations require intractable pain despite non-surgical treatment for at least 6 weeks and associated neurologic deficit. The regulations for fusion surgeries combined with decompression for cervical myelopathy are neurologic deficits attributed to this diagnosis. The surgical standard of care for patients with lumbar disc herniation in Korea is lumbar discectomy in case of the patients with intractable pain or neurologic deficits despite non-surgical treatment that lasted for at least 12 weeks. Regarding lumbar fusion for lumbar disc herniation, these regulations additionally require recurrent lumbar disc herniation, foraminal lumbar disc herniation, or lumbar instability combined with lumbar radiculopathy. The surgical standard of care for patients with degenerative spondylolisthesis or spinal stenosis in Korea is posterior lumbar decompression in the case of the patients with no improvement of symptoms despite non-surgical treatment that lasts for at least 12 weeks. Concerning lumbar fusion for degenerative spondylolisthesis or spinal stenosis, these regulations additionally require findings of foraminal stenosis or lumbar instability combined with degenerative spondylolisthesis or spinal stenosis. Therefore, the requirements of the Korean National Health Insurance Corporation were considered as the surgical indications for patients in this cohort.

### Confounding factors

In the current study, age, gender, the presence of diabetes, associated comorbidities, and hospital types were considered as potential confounding factors. Medical comorbidities were assessed according to the ICD-10, proposed by Quan et al. [15] If there were more than 4 distinct primary or secondary diagnoses in 2012, the patients were regarded as having associated medical comorbidities [16, 17]. Diabetes was analyzed separately because it is known as the risk factor for reoperation that increases complication rates and inhibits functional recovery [16, 18].

In Korea, the law designates types of the hospital [16]. General hospitals should have at least seven departments including internal medicine, general surgery, obstetrics and gynecology, pediatrics, diagnostic radiology, anesthesiology, pathology, and laboratory medicine as well as at least one board-certified doctor in each department with > 99 beds. Tertiary-referral hospitals are differentiated from general hospitals by having at least 20 departments. Also, they should have residency programs, at least 5 operating rooms, and various diagnostic tools such as computed tomography, magnetic resonance imaging, electromyography, angiography, gamma camera radiography, Holter cardiac monitoring, etc. Hospitals are a healthcare center that does not have essential departments mentioned above or those with 30—99 beds. Private clinics have < 30 beds.

### Statistical analysis

Time to event (reoperation) survival analysis was performed. The primary endpoint was reoperation during the follow-up period. Presence of any procedure codes including the aforementioned procedure codes registered after the index procedure code was identified as reoperation. Since later interventions may not have portrayed the natural history that occurred after fusion operations, the third and subsequent reoperation events were excluded from the cumulative operation rates. January 1, 2012, the first date in our data collection period, and June 30, 2017, the last date, were used if the latter date was not available. Therefore, the minimal follow-up period is four and a half years (from January 1, 2013, to June 30, 2017). Reoperation rates were analyzed early (before 1 year postoperatively) or late (after 1 year postoperatively) period of follow-up. Chi-square tests or t-test was used to compare the baseline characteristics of the subjects. Statistical analysis for comparison between the two groups was performed with Cox proportional hazards regression modeling. Statistical analysis for the comorbidities according to hospital types was performed with 4-sample proportional test. Data were analyzed by the Statistical Analysis System (SAS) software version 6.1 (SAS Institute, Inc., Cary, NC, USA). A *p-*value of < 0.05 was considered statistically significant.

## Results

Lumbar fusion surgeries were more commonly encountered in our cohort than cervical fusion surgeries (71.98 and 28.02%, respectively, Table 1). The mean patient age was 59.47 ± 11.93 years; 56.08% were women. Age, gender, the presence of diabetes, associated comorbidities, hospital types, and surgical approaches were different between the two groups. The surgical approach was not considered a potential confounding factor because the anterior approach was found in 93.69% of cervical surgeries and a posterior approach was found in 97.83% of lumbar surgeries. The most common comorbidity of study population was the diabetes (39.94%) and the comorbidities in the single cohorts of hospital types were not different except the diabetes (Table 2).

**Table 1:** The characteristics of the study population

Number (%)	All patients	Cervical fusion	Lumbar fusion	*P*
	42,060	11,784 (28.02%)	30,276 (71.98%)	
Age (years)				** < 0.0001**
20–29	514 (1.22%)	206 (1.75%)	308 (1.02%)	
30–39	1,933 (4.60%)	1,092 (9.27%)	841 (2.78%)	
40–49	5,905 (14.04%)	3,455 (29.32%)	2,450 (8.09%)	
50–59	11,770 (27.98%)	4,032 (34.22%)	7,738 (25.56%)	
60–69	12,247 (29.12%)	1,928 (16.36%)	10,319 (34.08%)	
≥ 70	9,691 (23.04%)	1,071 (9.09%)	8,620 (28.47%)	
Mean age (SD)	59.47 ± 11.93	52.74 ± 11.36	62.09 ± 11.08	**0.0012**
Gender, female, n	23,587 (56.08%)	4,318 (36.64%)	19,269 (63.64%)	** < 0.0001**
Diabetes, n	16,610 (39.49%)	4,067 (34.51%)	12,543 (41.43%)	** < 0.0001**
Associated comorbidities, n	20,268 (48.19%)	4,937 (41.90%)	15,331 (50.64%)	** < 0.0001**
Hospital types				
Tertiary-referral hospital	11,179 (25.58%)	3,711 (31.49%)	7,468 (24.67%)	** < 0.0001**
General hospital	12,507 (29.74%)	3,142 (26.66%)	9,365 (30.93%)	
Hospital	17,956 (42.69%)	4,842 (41.09%)	13,114 (43.31%)	
Clinic	418 (0.99%)	89 (0.76%)	329 (1.09%)	
Surgical approaches				** < 0.0001**
Anterior	11,698 (27.81%)	11,040 (93.69%)	658 (2.17%)	
Posterior	30,362 (72.19%)	744 (6.31%)	29,618 (97.83%)

**Table 2:** Comorbidities of study population and those according to hospital types

	All patients (*n* = 42,060)	Tertiary-referral hospital (*n* = 11,179)	General hospital (*n* = 12,507)	Hospital (*n* = 17,956)	Clinic (*n* = 418)	*P*
Diabetes	16,609 (39.49%)	4,200 (37.58%)	4,702 (37.59%)	7,527 (41.92%)	180 (43.06%)	** < 0.001**
Osteoporosis	278 (0.66%)	76 (0.68%)	80 (0.64%)	118 (0.66%)	4 (0.96%)	0.8711
Myocardial infarction	2,326 (5.53%)	629 (5.63%)	688 (5.50%)	994 (5.54%)	15 (3.59%)	0.3565
Congestive heart failure	307 (0.73%)	82 (0.73%)	89 (0.71%)	132 (0.74%)	4 (0.96%)	0.9476
Peripheral vascular disease	349 (0.83%)	93 (0.83%)	100 (0.80%)	152 (0.85%)	4 (0.96%)	0.9632
Cerebrovascular disease	1,077 (2.56%)	277 (2.48%)	322 (2.57%)	470 (2.62%)	8 (1.91%)	0.7410
Dementia	135 (0.32%)	36 (0.32%)	42 (0.34%)	56 (0.34%)	1 (0.28%)	0.9743
Chronic pulmonary disease	3,987 (9.48%)	1,063 (9.51%)	1,188 (9.50%)	1,700 (9.47%)	36 (8.61%)	0.9432
Rheumatic disease	437 (1.04%)	120 (1.07%)	128 (1.02%)	186 (1.04%)	3 (0.72%)	0.9010
Peptic ulcer disease	1,195 (2.84%)	306 (2.74%)	361 (2.89%)	513 (2.86%)	15 (3.59%)	0.7075
Hemiplegia or paraplegia	227 (0.54%)	63 (0.56%)	65 (0.52%)	98 (0.55%)	1 (0.24%)	0.8189
Renal disease	454 (1.08%)	126 (1.13%)	130 (1.04%)	195 (1.09%)	4 (0.96%)	0.9215
Any malignancy	109 (0.26%)	26 (0.23%)	36 (0.29%)	46 (0.26%)	1 (0.24%)	0.8694
Liver disease	5,897 (14.02%)	1,586 (14.19%)	1,742 (13.93%)	2,520 (14.03%)	49 (11.72%)	0.5358
AIDS/HIV	0 (0.00%)	0 (0.00%)	0 (0.00%)	0 (0.00%)	0 (0.00%)	NA

The total reoperation rate was 2.62% during the entire follow-up period. The reoperation rate of the cervical fusion group was 2.33% and the lumbar fusion group was 2.74% (Table 3). The anatomical region of the lumbar spine, the presence of diabetes, associated comorbidities, and hospital types were detected to be significant confounding factors by Cox regression analysis (Table 4).

**Table 3:** Reoperation rates of fusion surgeries according to anatomic regions

Postoperative time	Cervical fusion (n)	Cumulative cervical fusion (n)	Cumulative cervical reoperation rate	Lumbar fusion (n)	Cumulative lumbar fusion (n)	Cumulative lumbar reoperation rate
< 1 month	15	15	0.13%	66	66	0.22%
1–2 months	7	22	0.19%	21	87	0.29%
2–3 months	7	29	0.25%	10	97	0.32%
3–6 months	13	42	0.36%	33	130	0.43%
6 mo–1 yr	9	51	0.43%	23	153	0.51%
1–2 yr	28	79	0.67%	93	246	0.81%
2–3 yr	89	168	1.43%	248	494	1.63%
3–4 yr	78	246	2.09%	230	724	2.39%
≥ 4 yr	28	274	2.33%	105	829	2.74%

**Table 4:** Cumulative reoperation rates of fusion surgeries according to anatomic regions during the entire follow up period

Entire period (*n* = 42,060)	Unadjusted value			Adjusted value		
	*P*	HR	95% CI	*p*	HR	95% CI
Region						
Cervical fusion		1.000			1.000	
Lumbar fusion	**0.0134**	1.188	(1.036, 1.362)	**0.0275**	1.167	(1.017,1.339)
Age (years)						
20–29		1.000				
30–39	0.7030	0.871	(0.430, 1.768)			
40–49	0.8510	0.940	(0.492, 1.796)			
50–59	0.4561	1.271	(0.677, 2.387)			
60–69	0.1760	1.543	(0.823, 2.892)			
≥ 70	0.1671	1.559	(0.830, 2.929)			
Gender						
Male		1.000				
Female	0.7845	0.9840	(0.873, 1.108)			
Diabetes						
Yes	**0.0168**	1.157	(1.027, 1.303)			
No		1.000				
Associated comorbidities						
Yes	**0.0003**	1.241	(1.103, 1.397)	**0.0019**	1.208	(1.072,1.362)
No		1.000			1.000	
Hospital types						
Tertiary-referral hospital		1.000			1.000	
General hospital	**0.0113**	1.212	(1.044, 1.408)	**0.0455**	1.166	(1.003, 1.355)
Hospital	**0.0038**	0.801	(0.689, 0.931)	**0.0014**	0.782	(0.673, 0.910)
Clinic	0.3403	0.711	(0.352, 1.434)	0.3434	0.712	(0.353, 1.437)

**Fig. 2:**
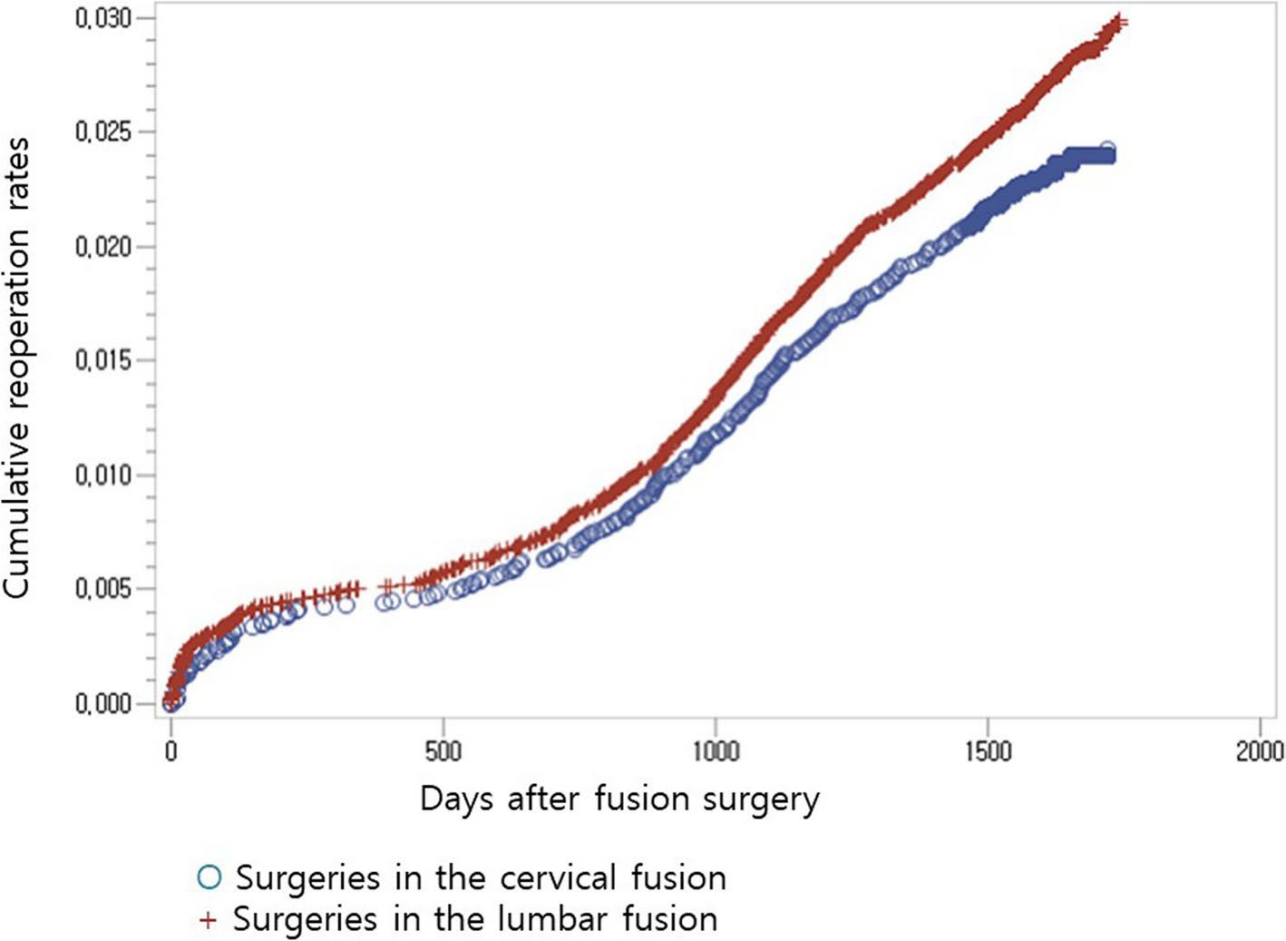
Cumulative reoperation rates of fusion surgeries according to anatomical regions for the entire follow-up period

After adjusting for these confounders, the reoperation rate was higher in the patients who underwent lumbar fusion surgery than in those who underwent cervical fusion surgery during the entire follow up period (lumbar fusion: *p* = 0.0275, hazard ratio = 1.167, 95% confidence interval [CI] 1.017–1.339, Fig. 2, Table 4). The associated comorbidities, and hospital types were found to significantly affect the risk for reoperations (associated comorbidities: *p* = 0.0019, hazard ratio = 1.208, 95% confidence interval [CI] 1.072–1.362; general hospital: *p* = 0.0455, hazard ratio = 1.166, 95% confidence interval [CI] 1.003–1.355; hospital: *p* = 0.0014, hazard ratio = 0.782, 95% confidence interval [CI] 0.673–0.910, Table 4). Hospital type of general hospitals has more reoperations than that of tertiary-referral hospitals have, and the type of hospitals have fewer reoperations than that of tertiary-referral hospitals have.

In the early period, there was no difference in reoperation rates between the two groups (Table 5). However, in the late period, the reoperation rate was higher in the patients who underwent lumbar fusion surgery than in the patients who underwent cervical fusion surgery (lumbar fusion: *p* = 0.0468, hazard ratio = 1.166, 95% confidence interval [CI] 1.002–1.358, Table 6). The associated comorbidities were found to significantly affect the risk for reoperations (associated comorbidities: *p* = 0.0002, hazard ratio = 1.286, 95% confidence interval [CI] 1.127–1.467, Table 6).

**Table 5:** Cumulative reoperation rates of fusion surgeries according to anatomic regions during the early period

Early period (*n* = 206)	Unadjusted value		
	*P*	HR	95% CI
Region			
Cervical fusion		1.000	
Lumbar fusion	0.7947	1.043	(0.758, 1.436)
Age (years)			
20–29		1.000	
30–39	0.3929	0.542	(0.133, 2.208)
40–49	0.5079	1.457	(0.478, 4.442)
50–59	0.7599	1.173	(0.421,3.267)
60–69	0.8674	1.090	(0.397, 2.994)
≥ 70	0.7939	1.144	(0.416, 3.144)
Gender			
Male		1.000	
Female	0.8193	0.968	(0.733, 1.279)
Diabetes			
Yes	0.6594	0.938	(0.704, 1.248)
No		1.000	
Associated comorbidities			
Yes	0.4081	0.890	(0.675, 1.173)
No		1.000	
Hospital types			
Tertiary-referral hospital		1.000	
General hospital	**0.0050**	1.610	(1.155, 2.244)
Hospital	0.0865	0.704	(0.472, 1.052)
Clinic	0.7124	0.689	(0.095, 4.993)

**Table 6:** Cumulative reoperation rates of fusion surgeries according to anatomic regions during the late period

Late period (*n* = 41,856)	Unadjusted value			Adjusted value		
	*P*	HR	95% CI	*p*	HR	95% CI
Region						
Cervical fusion		1.000			1.000	
Lumbar fusion	**0.0225**	1.193	(1.025, 1.388)	**0.0468**	1.166	(1.002, 1.358)
Age (years)						
20–29		1.000				
30–39	0.5879	1.275	(0.529, 3.071)			
40–49	0.4621	1.363	(0.597, 3.110)			
50–59	0.1674	1.769	(0.787, 3.976)			
60–69	0.0701	2.110	(0.941, 4.735)			
≥ 70	0.0946	1.997	(0.888, 4.492)			
Gender						
Male		1.000				
Female	0.9412	0.995	(0.872, 1.135)			
Diabetes						
Yes	**0.0025**	1.225	(1.074, 1.398)			
No		1.000				
Associated comorbidities						
Yes	** < 0.0001**	1.300	(1.140, 1.482)	**0.0002**	1.286	(1.127, 1.467)
No		1.000			1.000	
Hospital types						
Tertiary-referral hospital		1.000				
General hospital	0.2335	1.108	(0.936, 1.311)			
Hospital	0.0848	0.866	(0.735, 1.020)			
Clinic	0.4762	0.761	(0.359, 1.613)			

## Discussion

The purpose is to compare reoperations after fusion surgeries for degenerative spinal diseases according to cervical and lumbar regions.

The reoperation rate was higher in the patients who underwent lumbar fusion surgery than in the patients who underwent cervical fusion surgery during the entire follow up period. A similar pattern was found after 1 year postoperatively. However, before 1 year postoperatively, there was no difference in reoperation rates between the two groups. Associated comorbidities and hospital type were noted to be risk factors for reoperation.

Cervical revisional fusion was 3.2 to 3.4% after the index cervical fusions and lumbar revisional fusion was 5.4 to 6.8% after the index lumbar fusion between 2002 and 2009 based on the United States Nationwide Inpatient Sample [19]. They found that the numbers of revisional fusion surgeries in the cervical and lumbar spines increased annually, but they did not compare the revisional rates between the cervical and lumbar fusion surgery groups in a statistical manner [19]. The nonunion rates of cervical fusion surgeries were 1.6% and those of lumbar fusion surgeries were 2.0% based on the data of a spine registry of 3.401cases between 2009 and 2011 [20]. However, they did not compare the nonunion rate of cervical fusion surgeries with that of lumbar fusion surgeries in a statistical manner, too [20].

The reoperation rate was higher in the patients who underwent lumbar fusion surgery than in the patients who underwent cervical fusion surgery in the current study. It might be due to the differences in surgical approaches related to the anatomical differences. The posterior surgical approach was found in 97.83% of lumbar surgeries and the anterior surgical approach was used in 93.69% of cervical fusion surgeries in the current study. In the study with administrative data for the lumbar stenosis patients who had undergone the operations with a follow-up of 10 years, there was no difference in reoperation rates between the anterior fusion surgeries and the posterior fusion surgeries [21]. In contrast, in the case of cervical degenerative diseases, the patients with anterior fusions had lower complication rates than those with posterior fusions in the study with administrative data with a follow-up of 10 years [22]. The patients with anterior cervical fusions had lower reoperation rates than those with posterior cervical fusions in the study with administrative data with a follow-up of 5 years, too [23]. The adjacent segmental pathologies requiring reoperations were less common in the patients with anterior fusion surgeries than those with posterior fusion surgeries in the survivorship analysis of 1,358 patients who had undergone the cervical spine operations with a follow-up of 4 years [24]. The reoperations were less common in the group of anterior fusion surgeries than in the group of posterior fusion surgeries in the study with the patients who had undergone cervical fusion surgeries for the degenerative cervical pathologies in more than three disc levels [25]. However, Derman et al. queried the New York State’s all-payer health care database of 87.042 patients from 1997 to 2012 and found that the risk of revision surgeries was higher for anterior cervical fusion surgeries compared with posterior cervical fusion surgeries with the follow-up of 16 years [26]. They insisted that at the longer follow-up the cumulative revision surgeries for anterior approaches began to exceed that of posterior approaches [26]. In addition, the difference of reoperation rates between both groups might be due to the endogenous reason that the lumbar spine has a higher weight and more local pressure than the cervical spine to have more altered biomechanical forces near a previous fusion site [27]. The biomechanical effect of anterior cervical discectomy and fusion (ACDF) surgery on the adjacent levels was smaller compared to that of posterior lumbar fusion surgery [28]. The small bone mineral density (BMD) decreased in adjacent vertebrae following ACDF surgery compared to large BMD loss of posterior lumbar fusion surgery [28].

The associated comorbidities were found to significantly affect the risk of reoperations. Similarly to the current study, the associated comorbidities had a greater chance of occurrence of revision spinal fusion after all spinal fusions including cervical, thoracic and lumbar spines based on the United States Nationwide Inpatient Sample from 2002 to 2009 [19]. The associated comorbidity was a risk factor for reoperation in patients with lumbar disc herniation in the study based on the Korean administrative data, too [16].

General hospitals have more reoperations than tertiary-referral hospitals have. There have been controversies over the size of the hospital. Hospital type did not affect complications and mortality in patients who underwent cervical corpectomy in the administrative data-based study [29]. However, the complication rate is 6.1% when the hospital size is small and 8.8% when the hospital size is large in patients with cervical spondylotic myelopathy [30]. In contrast, reoperation was more common in small private clinics than large hospitals for patients with lumbar disc herniation and lumbar stenosis [16, 31]. The controversies over the size of the hospital type might be explained by the fact that reoperation rates be influenced by not only comorbidities but also by several other confounding factors of the surgical skill and experience of surgeons, the medical facilities, and the patients’ economic capabilities and preferences for reoperations. In the current study, the comorbidities in the single cohorts of hospital types were not different except diabetes.

There was no difference in reoperation rates between the two groups before 1 year postoperatively but the reoperation rate was different between them after 1 year postoperatively. It might be explained by the fact that it takes a long time to achieve bony fusion. Therefore, it might be because it takes a long time to make complications of adjacent segmental diseases which are related to bony fusion to reach to reoperations [32].

Similarly with the current study, the study based on the United States Nationwide Inpatient Sample had the patients characteristics that those which underwent lumbar spinal fusion surgeries had a higher proportion of the patients older than 65 years (32.4 versus 20.4%), more women (55.4 versus 51.0%), and more chronic associated comorbidities (4.0 versus 3.6%) than those which underwent cervical spinal fusion surgeries [19].

As with any study, our investigation has several limitations. First, there was no information about the clinical symptoms, signs, and radiologic findings. The reason for the reoperations is an important key to understand the failure of spinal fusion surgery. The current study is based on the administrative data. Therefore, we did not provide information about what caused the reoperations. We could only give a general outline of reoperations. The current study may give little help in understanding the medical cause-and–effect relationship but some help to understand the overall clinical practice based on the large administrative data, which could not be elucidated by the specific clinical information. Second, we did not analyze the data for the patients who underwent decompression alone. We have a plan to do it in the future. Third, the posterior surgical approach was found in 97.83% of lumbar surgeries and the anterior surgical approach was used in 93.69% of cervical fusion surgeries in the current study. It could be a selection bias. However, we had inspected all of the spinal fusion surgeries which had been performed in 2012 in the Republic of Korea without selection. Despite these limitations, to the best of our knowledge, this study represents the first population-based analysis of the reoperation rates after fusion surgeries according to cervical and lumbar regions.

## Conclusions

The incidence of reoperation was higher in the patients who underwent lumbar fusion surgery than in the patients who underwent cervical fusion surgery for degenerative spinal diseases. This information could help the surgeons and the patients discuss the clinical strategies to solve their clinical problems, especially dealing with the patients with concurrent spinal pathologies in the cervical and lumbar spines.

## Data Availability

All of the data supporting findings are contained within the manuscript. Disaggregated data cannot be made publicly available because of ethical restrictions. Please contact the corresponding author.

## References

[1] Jacobs B, Ghelman B, Marchisello P (1990). Coexistence of cervical and lumbar disc disease. Spine (Phila Pa 1976).

[2] Teng P, Papatheodorou C (1964). Combined cervical and lumbar spondylosis. Arch Neurol.

[3] Choudhury AR, Taylor JC (1980). The cervicolumbar syndrome. Ann R Coll Surg Engl.

[4] Irmola TM, Hakkinen A, Jarvenpaa S, Marttinen I, Vihtonen K, Neva M (2018). Reoperation rates following instrumented lumbar spine fusion. Spine (Phila Pa 1976).

[5] Nemani VM, Aichmair A, Taher F, Lebl DR, Hughes AP, Sama AA, Cammisa FP, Girardi FP (2014). Rate of revision surgery after stand-alone lateral lumbar interbody fusion for lumbar spinal stenosis. Spine (Phila Pa 1976).

[6] Radcliff K, Spivak J, Darden B, Janssen M, Bernard T, Zigler J (2018). Five-year reoperation rates of 2-level lumbar total disk replacement versus fusion: results of a prospective, randomized clinical trial. Clin Spine Surg.

[7] Lubelski D, Healy AT, Silverstein MP, Abdullah KG, Thompson NR, Riew KD, Steinmetz MP, Benzel EC, Mroz TE (2015). Reoperation rates after anterior cervical discectomy and fusion versus posterior cervical foraminotomy: a propensity-matched analysis. Spine J.

[8] van Eck CF, Regan C, Donaldson WF, Kang JD, Lee JY (2014). The revision rate and occurrence of adjacent segment disease after anterior cervical discectomy and fusion: a study of 672 consecutive patients. Spine (Phila Pa 1976).

[9] Lee SH, Kim KT, Suk KS, Lee JH, Shin JH, So DH, Kwack YH (2010). Asymptomatic cervical cord compression in lumbar spinal stenosis patients: a whole spine magnetic resonance imaging study. Spine (Phila Pa 1976).

[10] Okada E, Matsumoto M, Fujiwara H, Toyama Y (2011). Disc degeneration of cervical spine on MRI in patients with lumbar disc herniation: comparison study with asymptomatic volunteers. Eur Spine J.

[11] Lawrence JS (1969). Disc degeneration. Its frequency and relationship to symptoms. Ann Rheum Dis.

[12] Burkhardt BW, Simgen A, Wagenpfeil G, Hendrix P, Reith W, Oertel JM (2020). Adjacent segment disease following anterior cervical fusion and the presence of surgery for lumbar disc herniation and surgery at the musculoskeletal joints: are they related?. Spine J.

[13] Park MS, Ju YS, Moon SH, Kim TH, Oh JK, Makhni MC, Riew KD (2016). Reoperation rates after surgery for degenerative cervical spine disease according to different surgical procedures: national population-based cohort study. Spine (Phila Pa 1976).

[14] Park MS, Ju YS, Moon SH, Kim TH, Oh JK, Makhni MC, Riew KD (2016). Reoperation rates after anterior cervical discectomy and fusion for cervical spondylotic radiculopathy and myelopathy: a national population-based study. Spine (Phila Pa 1976).

[15] Quan H, Sundararajan V, Halfon P, Fong A, Burnand B, Luthi JC, Saunders LD, Beck CA, Feasby TE, Ghali WA (2005). Coding algorithms for defining comorbidities in ICD-9-CM and ICD-10 administrative data. Med Care.

[16] Kim CH, Chung CK, Park CS, Choi B, Kim MJ, Park BJ (2013). Reoperation rate after surgery for lumbar herniated intervertebral disc disease: nationwide cohort study. Spine (Phila Pa 1976).

[17] Hu RW, Jaglal S, Axcell T, Anderson G (1997). A population-based study of reoperations after back surgery. Spine (Phila Pa 1976).

[18] Takahashi S, Suzuki A, Toyoda H, Terai H, Dohzono S, Yamada K, Matsumoto T, Yasuda H, Tsukiyama K, Shinohara Y (2013). Characteristics of diabetes associated with poor improvements in clinical outcomes after lumbar spine surgery. Spine (Phila Pa 1976).

[19] Rajaee SS, Kanim LE, Bae HW (2014). National trends in revision spinal fusion in the USA: patient characteristics and complications. Bone Joint J.

[20] Guppy KH, Paxton EW, Harris J, Alvarez J, Bernbeck J (2014). Does bone morphogenetic protein change the operative nonunion rates in spine fusions?. Spine (Phila Pa 1976).

[21] Jung JM, Chung CK, Kim CH, Choi Y, Kim MJ, Yim D, Yang SH, Lee CH, Hwang SH, Kim DH (2020). The long-term reoperation rate following surgery for lumbar stenosis: a nationwide sample cohort study with a 10-year follow-up. Spine (Phila Pa 1976).

[22] Wang MC, Chan L, Maiman DJ, Kreuter W, Deyo RA (2007). Complications and mortality associated with cervical spine surgery for degenerative disease in the United States. Spine (Phila Pa 1976).

[23] King JT, Abbed KM, Gould GC, Benzel EC, Ghogawala Z (2009). Cervical spine reoperation rates and hospital resource utilization after initial surgery for degenerative cervical spine disease in 12,338 patients in Washington State. Neurosurgery.

[24] Lee JC, Lee SH, Peters C, Riew KD (2014). Risk-factor analysis of adjacent-segment pathology requiring surgery following anterior, posterior, fusion, and nonfusion cervical spine operations: survivorship analysis of 1358 patients. J Bone Joint Surg Am.

[25] Cole T, Veeravagu A, Zhang M, Azad TD, Desai A, Ratliff JK (2015). Anterior versus posterior approach for multilevel degenerative cervical disease: a retrospective propensity score-matched study of the MarketScan database. Spine (Phila Pa 1976).

[26] Derman PB, Lampe LP, Hughes AP, Pan TJ, Kueper J, Girardi FP, Albert TJ, Lyman S (2016). Demographic, clinical, and operative factors affecting long-term revision rates after cervical spine arthrodesis. J Bone Joint Surg Am.

[27] Tobert DG, Antoci V, Patel SP, Saadat E, Bono CM (2017). Adjacent segment disease in the cervical and lumbar spine. Clin Spine Surg.

[28] Salzmann SN, Okano I, Miller CO, Chiapparelli E, Reisener MJ, Amini DA, Winter F, Shue J, Carrino JA, Sama AA et al. The cervical spine demonstrates less postoperative bone loss than the lumbar spine. J Orthop Res. 2021. 10.1002/jor.25069. 10.1002/jor.25069 33914982

[29] Boakye M, Patil CG, Ho C, Lad SP (2008). Cervical corpectomy: complications and outcomes. Neurosurgery.

[30] Boakye M, Patil CG, Santarelli J, Ho C, Tian W, Lad SP (2008). Cervical spondylotic myelopathy: complications and outcomes after spinal fusion. Neurosurgery.

[31] Kim CH, Chung CK, Park CS, Choi B, Hahn S, Kim MJ, Lee KS, Park BJ (2013). Reoperation rate after surgery for lumbar spinal stenosis without spondylolisthesis: a nation-wide cohort study. Spine J.

[32] Martin BI, Mirza SK, Comstock BA, Gray DT, Kreuter W, Deyo RA (2007). Reoperation rates following lumbar spine surgery and the influence of spinal fusion procedures. Spine (Phila Pa 1976).

